# Cortical thickness in the right medial frontal gyrus predicts planning performance in healthy children and adolescents

**DOI:** 10.3389/fpsyg.2023.1196707

**Published:** 2023-09-19

**Authors:** Kathrin Kollndorfer, Astrid Novak, Karl-Heinz Nenning, Florian Ph S. Fischmeister, Rainer Seidl, Georg Langs, Gregor Kasprian, Daniela Prayer, Lisa Bartha-Doering

**Affiliations:** ^1^Department of Biomedical Imaging and Image-guided Therapy, Medical University of Vienna, Developmental and Interventional Imaging (DIN) Lab, Vienna, Austria; ^2^Department of Pediatrics and Adolescent Medicine, Medical University Vienna, Vienna, Austria; ^3^Department of Biomedical Imaging and Image-guided Therapy, Medical University of Vienna, Computational Imaging Research Lab (CIR), Vienna, Austria; ^4^Center for Biomedical Imaging and Neuromodulation, Nathan S. Kline Institute for Psychiatric Research, Orangeburg, NY, United States; ^5^Institute of Psychology, University of Graz, Graz, Austria; ^6^BioTechMed-Graz, Graz, Austria; ^7^Department of Biomedical Imaging and Image-guided Therapy, Medical University of Vienna, Vienna, Austria

**Keywords:** executive functions, developmental imaging, planning, cortical thickness, medial frontal gyrus

## Abstract

The ability to plan is an important part of the set of the cognitive skills called “executive functions.” To be able to plan actions in advance is of great importance in everyday life and constitutes one of the major key features for academic as well as economic success. The present study aimed to investigate the neuroanatomical correlates of planning in normally developing children, as measured by the cortical thickness of the prefrontal cortex. Eighteen healthy children and adolescents underwent structural MRI examinations and the Tower of London (ToL) task. A multiple regression analysis revealed that the cortical thickness of the right caudal middle frontal gyrus (cMFG) was a significant predictor of planning performance. Neither the cortical thickness of any other prefrontal area nor gender were significantly associated with performance in the ToL task. The results of the present exploratory study suggest that the cortical thickness of the right, but not the left cMFG, is positively correlated with performance in the ToL task. We, therefore, conclude that increased cortical thickness may be more beneficial for higher-order processes, such as information integration, than for lower-order processes, such as the analysis of external information.

## Introduction

The ability to plan actions in advance is one of the basic components of problem-solving and is essential in everyday life. Planning comprises the mental modeling of actions, as well as the anticipation of potential consequences prior to the realization of the action in the real world (Goel and Grafman, 1995; Ward and Morris, 2005). Lesion studies have identified the dorsolateral prefrontal cortex (DLPFC) as a crucial brain area for planning in adults (Lazeron et al., 2000; Cazalis et al., 2003; Newman et al., 2003). Functional imaging studies in adults have underlined the important role of the DLPFC in both hemispheres in planning actions [for review see (Nitschke et al., 2017)]. However, findings in patients with brain lesions provided inconsistent results (Anderson et al., 2002; Jacobs and Anderson, 2002; Andrews et al., 2014). Previous research indicates different contributions by the left and right hemispheres at different stages of the planning process in adults (Kaller et al., 2011).

Planning actions plays a major role in the development and coordination of an appropriate response to the environment. Previous research has indicated that planning abilities undergo substantial development early in life (Klahr and Robinson, 1981; Kaller et al., 2008; McCormack and Atance, 2011). However, the development of planning abilities continues until early adulthood (Albert and Steinberg, 2011). Although an increasing number of studies have investigated the development of planning function from early childhood to adulthood, we still lack knowledge about the role of the DLPFC throughout different developmental stages. Most neuroimaging studies about planning function were performed in children with focal brain lesions. Jacobs and Anderson (2002) found that children with focal lesions involving the right prefrontal area had the most severe problems with self-regulation. The authors, therefore, claim that the right prefrontal regions may play a major role in the development of executive functions, in general, and planning abilities, in particular. The development of the planning function has gained increased attention within the last several decades. A rapidly growing number of behavioral studies were performed in normally developing children [e.g., (Welsh et al., 1991; Asato et al., 2006; Kaller et al., 2008; Ni et al., 2011)], for various pathologies, such as autism spectrum disorder (Robinson et al., 2009), specific language impairment [SLI; (Roello et al., 2015)], or attention deficit hyperactivity disorder [ADHD; (Sonuga-Barke et al., 2002)]. However, little is known about the structural brain parameters related to planning abilities in normally developing children. We, therefore, aimed to study structural brain parameters in healthy children in relation to their planning abilities.

A relatively new parameter by which to measure neural substrates is cortical thickness (CTh). Previous studies suggest an association between cortical thickness and cognitive performance in various domains, ranging from general intelligence (Sowell et al., 2004; Karama et al., 2011; Menary et al., 2013; Burgaleta et al., 2014) to more specific abilities, such as working memory (Metzler-Baddeley et al., 2016), problem solving abilities (Squeglia et al., 2013), or visual perception (Poirel et al., 2014). The measurement of CTh is more specific compared to voxel based morphometry (VBM) (Hutton et al., 2009). VBM classifies brain volume via voxel-wise comparison of anatomical images into different tissue classes. Cortical volume is a product of cortical thickness and cortical surface area. According to previous research, these two parameters capture at least two distincts of genetic influences (Panizzon et al., 2009).

We aimed to investigate the relationship between brain structure, particularly cortical thickness, and planning abilities in healthy children, in order to gain more detailed information about the specific role of different prefrontal areas in the planning function in normally developing children and adolescents.

## Materials and methods

### Participants

Eighteen healthy children, 7 to 15 years of age (mean 10.61, SD 2.87), participated in this study. All children (7 female, 11 male) were native, monolingual speakers of German, with no history of any neurological or psychiatric disease and no clinical evidence of developmental delay. Included children had no history of traumatic brain injury. All children had normal or corrected-to-normal vision, normal hearing, and at least average performance intelligence (mean *z*-score 0.529, SD 15.91), as measured by the Hamburger-Wechsler-Intelligenztest für Kinder IV (HAWIK IV) (Petermann and Petermann, 2010). All children were right-handed, according to the Edinburgh Handedness Inventory (EHI) (Oldfield, 1971), and revealed a range of +70 to +100 (mean 96.67, SD 0.08). Children were recruited by flyer distribution and received a 30 € voucher to redeem in a book store.

### Ethical considerations

The study was performed in accordance with the Declaration of Helsinki (1964), and the study protocol was approved by the Ethics Committee of the Medical University of Vienna. All subjects and their legal representatives were informed about the aim of the study and gave written, informed consent prior to inclusion.

### Data acquisition

#### Tower of London

All participants underwent a standardized assessment of planning abilities using the German version of the Tower of London [ToL-D; (Tucha and Lange, 2004)]. In this test, three colored balls are arranged on three vertical pegs of different length. The balls must be moved one-by-one from an initial state to match a goal state depicted on a sheet. Problem complexity is manipulated by increasing the number of moves required and by altering the number of intermediate moves (i.e., where the ball has to be moved into a temporary position). Participants are instructed to plan the whole sequence of moves mentally before executing the sequence. Measures are taken of the number of trials solved in the minimum moves possible. In total, 20 problems were presented, with three, four, five and six minimum moves (five problems of each type). Each raw score is converted to an age-specific percentile. In addition, the average initial thinking time for three, four, five and six move problems were assessed separately.

#### Socioeconomic status

Socioeconomic status was evaluated based on a self-report of the household net income. In addition, the educational background from both, mother and father were assessed, as these factors were known to influence children’s cognitive development (Jackson et al., 2017).

#### Structural MRI imaging

All participants were scanned on a 3 T Siemens TIM Trio scanner (Siemens Medical Solutions, Erlangen, Germany) equipped with a high-performance gradient system to support fast, high-resolution, whole-brain echo-planar imaging. Three-dimensional, structural MRI scans were performed using an isocubic, magnetization-prepared, rapid gradient-echo (MPRAGE, T1-weighted, TE/TR = 4.21/2300 ms, slice thickness 1.10 mm, flip angle 9°) sequence.

### Data analysis

Subject-specific cortical thickness (CTh) was measured with FreeSurfer (v5.3.0; http://surfer.nmr.mgh.harvard.edu/). FreeSurfer performs automatic cortical and subcortical segmentation, and establishes an individual cortical surface model and its cortical folding patterns, providing measurements such as curvature, sulcal depth, and cortical thickness. For each cortical surface region, thickness was calculated as the distance between the white and pial surface (Fischl and Dale, 2000). To ensure comparability, subject-specific cortical surface models were registered to the FreeSurfer average cortical surface atlas (fsaverage5), where 72 regions of interest (36 per hemisphere) were defined based on the cortical neuroanatomical atlas by Desikan et al. (2006). For each region of interest, the CTh was averaged across all associated surface regions. For the present study, we restricted the analyses to the CTh of the prefrontal cortex of both hemispheres.

CTh in the prefrontal cortex was averaged across predefined areas: the rostral part of middle frontal gyrus (rMFG); the caudal part of the middle frontal gyrus (cMFG); the inferior frontal gyrus (IFG); the orbitofrontal cortex (medial and lateral orbitofrontal gyrus; OFC); and the anterior prefrontal cortex (frontal pole; aPFC) of the left and right hemisphere separately. As CTh is calculated from anatomical structures based on the Desikan et al. (2006) atlas, we thus refer to the MFG rather than the DLPFC in the present study. The DLPFC is a functional brain structure located anatomically in the middle frontal gyrus (MFG), i.e., in the lateral part of BA 9 and 46 (Cieslik et al., 2013).

Statistical analyses of behavioral data were conducted using the Statistical Package for the Social Sciences, version 24.0 (SPSS, Chicago, IL, United States). Due to the small sample size, a group comparison of ToL performance between genders was calculated using the Mann–Whitney U-test. A multiple regression analysis including both hemispheres and all selected regions was performed to investigate the impact of CTh in the prefrontal cortex on the performance of the ToL task. The alpha level was set at *α* = 0.05.

## Results

### Tower of London

**Figure 1:**
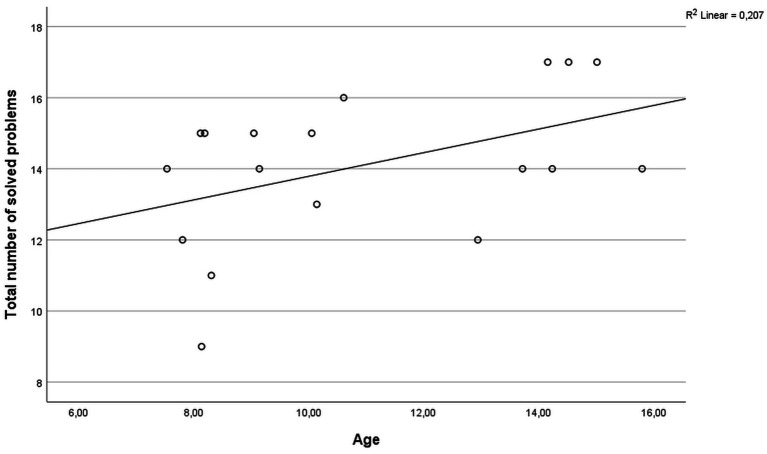
Scatter plot of the correlation between age and the total number of solved problems in the ToL task.

The performance of the participants on the ToL test was not associated with the educational background of the parents (mother: *r* = −0.121, *p* = 0.631; father: *r* = 0.086, *p* = 0.744) or the socioeconomic status (household net income; *r* = 0.093, *p* = 0.723). All participants showed an intelligence level in a normal or above average range (*z*-scores ranging from −0.267 to 1.665). In the ToL task, two participants showed below average performance (*z*-scores < −1), nine participants had average performance (*z*-scores between −1 and 1), and seven participants performed above average (*z*-scores > 1). No statistically significant difference in ToL performance between genders was observed (*U* = 38.0, *p* = 0.964). The total number of solved problems was significantly correlated with age (*r* = 0.486, *p* = 0.041, Figure 1). Detailed results of task performance are presented in Table 1.

**Table 1:** Detailed results cognitive testing.

	All subjects Mean (SD)	Correlation with educational background *r* (value of *p*)		Correlation with SES^a^ *r* (value of *p*)	Differences by gender value of *p*
		Mother	Father		
Age in years	10.61 (2.87)				0.536
Handedness	0.97 (0.08)				0.860
Performance intelligence (*z*-score)	0.61 (0.55)	0.130 (0.608)	0.44 (0.081)	0.388 (0.124)	0.360
Tower of London					
Overall *z*-score	0.29 (0.94)	−0.104 (0.681)	0.079 (0.764)	0.093 (0.724)	0.964
Number of solved problems (mean)					
Total	14.11 (2.14)	−0.227 (0.365)	−0.004 (0.988)	0.169 (0.516)	0.328
3 moves	4.94 (0.24)	−0.108 (0.668)	−0.165 (0.527)	−0.126 (0.631)	0.791
4 moves	3.89 (0.76)	−0.169 (0.504)	0.035 (0.893)	−0.387 (0.125)	0.479
5 moves	3.06 (1.00)	0.026 (0.920)	0.086 (0.742)	0.397 (0.115)	0.375
6 moves	2.17 (1.25)	−0.307 (0.215)	−0.096 (0.715)	0.206 (0.428)	0.659
Average initial thinking time^b^					
3 moves	3.57 (1.99)	0.308 (0.214)	−0.395 (0.117)	−0.264 (0.306)	0.930
4 moves	4.89 (3.01)	−0.073 (0.773)	0.170 (0.515)	0.321 (0.208)	0.536
5 moves	7.12 (6.75)	0.067 (0.790)	0.096 (0.714)	0.187 (0.472)	0.536
6 moves	11.62 (8.68)	0.070 (0.782)	0.105 (0.690)	0.144 (0.582)	0.126

a Socioeconomic status measured by household net income per year.

b In seconds.

### Cortical thickness

Group means and standard deviations of CTh per area are presented in Table 2. The CTh of prefrontal brain areas did not significantly correlate with age or gender. CTh was also not correlated with parents’ educational background (*r* ranging from −0.391 to 0.284; all *p* > 0.109).

**Table 2:** Cortical thickness in mm in frontal areas.

	All subjects mean (SD)	Correlation with age *r* (value of *p*)	Correlation with gender *r* (value of *p*)	Correlation with educational background *r* (value of *p*)	
				Mother	Father
Left hemisphere					
rMFG	2.47 (0.21)	−0.202 (0.421)	0.147 (0.561)	−0.030 (0.907)	−0.019 (0.942)
cMFG	2.62 (0.23)	0.270 (0.279)	0.195 (0.437)	−0.253 (0.312)	0.049 (0.851)
IFG	2.77 (0.18)	−0.009 (0.973)	0.391 (0.109)	−0.178 (0.481)	0.156 (0.550)
OFC	2.79 (0.17)	−0.313 (0.206)	0.261 (0.295)	−0.099 (0.695)	0.151 (0.563)
aPFC	2.95 (0.35)	−0.105 (0.678)	0.039 (0.879)	−0.391 (0.109)	−0.304 (0.236)
Right hemisphere					
rMFG	2.40 (0.22)	−0.302 (0.223)	0.067 (0.793)	0.284 (0.253)	0.228 (0.378)
cMFG	2.57 (0.18)	0.223 (0.375)	0.103 (0.685)	−0.133 (0.600)	−0.197 (0.449)
IFG	2.80 (0.21)	0.004 (0.988)	0.055 (0.829)	−0.053 (0.834)	0.104 (0.692)
OFC	2.76 (0.28)	−0.193 (0.444)	0.261 (0.296)	0.063 (0.804)	0.246 (0.341)
aPFC	2.87 (0.35)	−0.238 (0.342)	0.196 (0.435)	−0.158 (0.531)	0.001 (0.998)

rMFG, middle frontal gyrus (rostral part); cMFG, middle frontal gyrus (caudal part); IFG, inferior frontal gyrus; OFC, medial and lateral orbitofrontal gyrus; aPFC, anterior prefrontal cortex (frontal pole).

### Regression analysis

**Figure 2:**
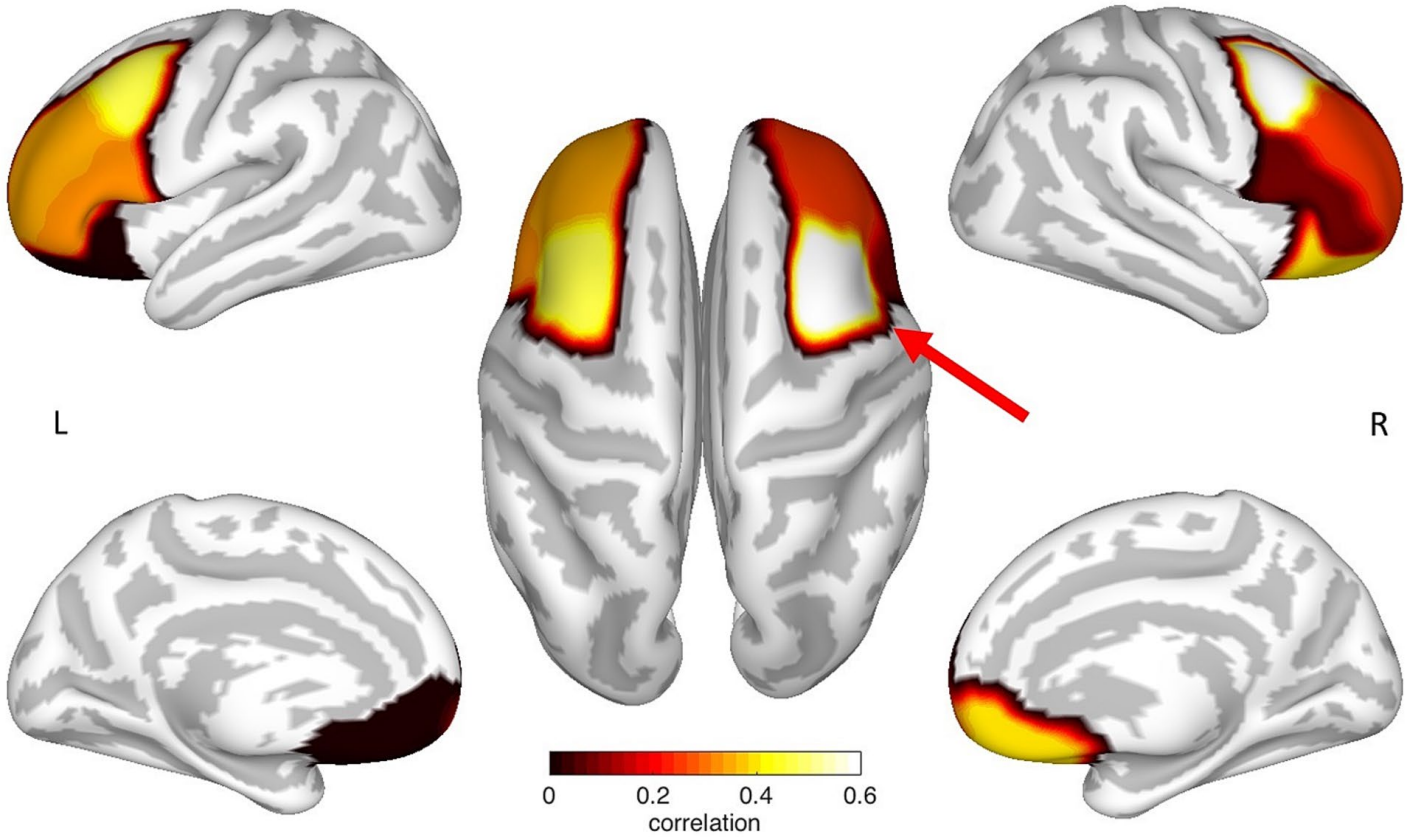
Mapping of the prefrontal ROI specific correlation coefficients between cortical thickness and ToL performance. Each correlation coefficient was mapped to the neuroanatomical location defined by the Desikan et al. (2006) atlas on a cortical surface model. Noticeably, the CTh of the left and the right cMFG showed an increased correlation, where only the right cMFG (red arrow) showed a significant relationship between cortical thickness and ToL performance. The figure represents non-thresholded results.

In a multiple regression analysis, the CTh of various areas of the prefrontal cortex was evaluated to predict the performance in the ToL task. Using stepwise iterations, the following measures were included as predictors in the model: age; gender; and the CTh of the OFC, the aPFC, the rMFG, the cMFG, and the IFG of the left and right hemisphere, respectively. The results of the regression analysis indicated one significant predictor for the ToL performance – the CTh of the right cMFG, which explained 34% of the variance (*R*^2^ = 0.379, *F*(1,16) = 9.766, *β* = 0.616, *p* = 0.007). Thus, this variable significantly predicted ToL performance (see Figure 2 and Table 3).

**Table 3:** Partial correlations of the regression analysis.

	Partial correlation with ToL task		
	*r*	Value of *p*	Beta
Age	−0.142	0.587	−0.115
Gender	−0.264	−307	−0.209
Left hemisphere			
rMFG	0.022	0.934	0.020
cMFG	0.045	0.863	0.052
IFG	0.046	0.861	0.041
OFC	−0.204	0.432	−0.167
aPFC	−0.190	0.465	−0.158
Right hemisphere			
rMFG	−0.029	0.913	−0.025
**cMFG**	**0.616**	**0.007**	**0.616**
IFG	−0.267	0.300	−0.235
OFC	0.089	0.734	0.085
aPFC	−0.017	0.950	−0.016

rMFG, middle frontal gyrus (rostral part); cMFG, middle frontal gyrus (caudal part); IFG, inferior frontal gyrus; OFC, medial and lateral orbitofrontal gyrus; aPFC, anterior prefrontal cortex (frontal pole); ToL, Tower of London.

Bold values represent significant correlations.

## Discussion

The main aim of the present study was to investigate the neuroanatomical correlates of planning abilities in normally developing children. We performed structural MRI and cognitive testing in 18 healthy children and adolescents, and investigated a possible association between cortical thickness (CTh) and the Tower of London (ToL) task. Regression analysis revealed CTh as a significant predictor for ToL performance. The CTh of the right cMFG was significantly higher in children with better planning ability. In contrast, neither the CTh of any other prefrontal brain area nor age or gender could predict the planning abilities of our study participants. To our knowledge, this is the first study to investigate the association between CTh and planning functions in normally developing children and adolescents.

Prefrontal areas have been reported to play a mediating role in planning functions in children and adults. The majority of fMRI studies that have investigated planning function in healthy human adults indicate bilateral activation of the DLPFC [e.g., (van den Heuvel et al., 2005; Boghi et al., 2006; Rasmussen et al., 2006; Fitzgerald et al., 2008; Kempton et al., 2011)]. These findings were supported by a meta-analysis of lesion studies in adult patients (Nitschke et al., 2017). In an event-related fMRI study using independently manipulated parameters, Kaller et al. (2011) showed that the involvement of the right and left DLPFC reflect different cognitive processes: whereas the activation of the left DLPFC was stronger for higher demands in goal hierarchy, the right DLPFC showed increased activation with higher demands in search depth. This finding was also supported in follow-up analyses (Nitschke et al., 2012; Ruh et al., 2012). The authors concluded that the left DLPFC is responsible for analyzing external information and is activated first. In a second step, the right DLPFC, which mainly contributes to the integration of information, and the monitoring of the planning process, is activated. These findings are of particular interest with respect to the present findings. Only the cortical thickness of the right cMFG was associated with the ToL performance, leading to the conclusion that a greater CTh is more beneficial for integrating information and less so for analyzing information. Similar to studies in adults, neuropediatric research has revealed that children with bilateral lesions showed the poorest performances in the ToL task. Children with lesions in the right prefrontal areas had more severe problems with self-regulation and goal-setting compared to children with left-lateralized lesions (Jacobs and Anderson, 2002). Thus, the right prefrontal cortex is assumed to play a crucial role in planning abilities. These results are in line with the findings of the present study, where the CTh of the right cMFG was a significant predictor of the ToL performance.

The development of planning is an ongoing process that starts in early childhood and continues until early adulthood. Previous research indicates that the improvement of ToL performance in adulthood is mainly mediated by the maturation of impulse control (Albert and Steinberg, 2011) and working memory capacity (Asato et al., 2006). The ongoing development of planning function until early adulthood is in line with neurobiological findings that have shown that the DLPFC is among the last brain regions to reach structural and functional maturity in late adolescence (Fuster, 2002; Giedd, 2008). The behavioral results of the present study, revealing an increase in the total number of solved problems with age, are in line with previous studies [e.g., (Luciana et al., 2009; Albert and Steinberg, 2011)], as well as with neurobiological theories that claim that maturation of the respective brain areas continues until late adolescence. In the present study no sex differences were observed. This was in line with a previous study by Unterrainer et al. (2015).

Previous research has discovered an association between CTh and various areas of cognitive performance. However, the interplay between cognitive performance and CTh appears to be complex, as neither a thicker nor thinner cortex can generally be interpreted as better or worse. CTh has been shown to correspond to genetic organization patterns throughout the entire life span (Fjell et al., 2015). An increase in CTh may be attributable to dendritic arborization [for review, see, e.g., (Chklovskii et al., 2004)], or learning-related synaptogenesis (Anderson et al., 2002). Based on these findings, it could be concluded that a thicker cortex corresponds to increased cognitive abilities. Indeed, a positive association between general intelligence and CTh was observed in children and young adults (Karama et al., 2011; Menary et al., 2013). Furthermore, in healthy children, an increased CTh was correlated with higher socioeconomic status and the educational status of the parents (Noble et al., 2015). Mackey et al. (2015) observed a positive correlation between increased CTh and higher income. In the present study sample, CTh was neither correlated with the parents’ educational background nor with household net income. One possible explanation of the missing association may be high mean educational status of the parents and the high mean income. Interestingly, many studies that have investigated specific cognitive abilities identified a negative correspondence between cognitive performance and CTh, e.g., for global vision (Poirel et al., 2014), mathematical performance (Chaddock-Heyman et al., 2015), or verbal learning (Squeglia et al., 2013). Cortical thinning with increasing age can partly be explained by intracortical myelination (Grydeland et al., 2013; Rowley et al., 2017; Fuhrmann et al., 2022). Another reason for decreasing of cortical thickness is synaptic pruning (Huttenlocher and Dabholkar, 1997; Petanjek et al., 2011). The reduction of synaptic density is a fundamental mechanism of neuroplasticity, underlying selective behavioral and even cognitive specialization (Edelman, 1993). Thus, both synaptic pruning, as well as synaptic expansion, seem to be necessary processes for the healthy development of the human brain (Poirel et al., 2014).

The assumption that both pruning and the development of new synapses are important mechanisms during healthy brain development is in line with findings from studies of various developmental or psychological disorders. Increased cortical thickness was observed in individuals with Down’s syndrome (Lee et al., 2016) and patients with Major Depressive Disorder (Foland-Ross et al., 2015; Fonseka et al., 2016). With respect to executive functions, increased CTh in the caudal inferior frontal gyrus was associated with poorer performance in a Go/NoGo Task in young adults with Attention Deficit Hyperactivity Disorder (ADHD) and in healthy controls (Newman et al., 2016). In contrast, decreased cortical thickness was observed in individuals with Autism Spectrum Disorder (Zielinski et al., 2014), in patients diagnosed with schizophrenia (Ehrlich et al., 2012), and in young adults with ADHD (Newman et al., 2016). Accelerated cortical thinning was also associated with substance abuse and heavy alcohol consumption during adolescence (Squeglia and Gray, 2016). The effect of substance abuse was not limited to the CTh of the developing brain after birth. Even prenatal exposure to substances such as alcohol or tobacco were related to lower cortical thickness during adolescence (Gautam et al., 2015). Based on previous research and the results of the present study, we conclude that both a lack of cortical thinning and extensive reduction of CTh is related to developmental, cognitive, and psychological dysfunction.

Interestingly, CTh was not correlated with the age or the gender of the participants. Previous studies have shown that CTh linearly decreases with age in healthy development (Sowell et al., 2004; Raznahan et al., 2010). It has been argued that the CTh may reflect underlying cellular and molecular processes, such as building and consolidation of synapses, as well as synaptic pruning (Burgaleta et al., 2014). The lack of a statistically significant interaction between age and CTh in the present study may be attributable to two reasons: first, the small sample size. Previous studies worked with large samples, measured twice, with a gap of approximately 2 years. Thus, these studies were based on the intraindividual development of very large study samples. Second, the highest decrease in CTh was observed in adolescence. A reduction of CTh between 0.02 and 0.04 mm per year has been observed, with a peak decrease in adolescence (Zhou et al., 2015). The present study comprised a sample of 18 healthy children and adolescents between 7 and 15 years. Thus, the majority of participants investigated in the present study were preadolescents, resulting in lower thinning rates.

A potential limitation of the present study is the small sample size of 18 normally developing children and adolescents, especially considering the relatively large age range of the participants. However, to the best of our knowledge, this is the first study to investigate lateralization of planning function, using cortical thickness measures, in normally developing children and adolescents. Furthermore, we investigated only right-handed participants, in order to avoid inconsistencies induced by the handedness of the participants. Future studies may investigate larger study samples and also include left-handed individuals. Furthermore, investigations of potential lateralization of other executive functions, such as problem-solving, reasoning, or working memory, using cortical thickness measures, would be of great interest.

## Conclusion

The results of the present study have shown that cortical thickness of the right, but not the left cMFG, is positively correlated with performance in the Tower of London task. The results of this exploratory study are in line with previous research on planning processes. Previous research has argued that the right and left DLPFC contribute to different cognitive demands during planning function: whereas the left DLPFC is responsible for analyzing external information, the right DLPFC contributes more to the integration of information and the monitoring of the planning process (Kaller et al., 2011). We, therefore, conclude that increased cortical thickness may be more beneficial for higher-order processes, such as integrating information, than for lower-order processes, such as analyzing external information.

## Data availability statement

The raw data supporting the conclusions of this article will be made available by the authors, without undue reservation.

## Ethics statement

The studies involving human participants were reviewed and approved by Ethics Committee Medical University of Vienna, Vienna, Austria. Written informed consent to participate in this study was provided by the participants’ legal guardian/next of kin.

## Author contributions

KK, RS, AN, and LB-D designed the study. AN and KK collected the data. K-HN, FF, GK, GL, and KK analysed the data. KK, DP, and LB-D wrote the manuscript. All authors contributed to the article and approved the submitted version.

## Funding

This work was funded by the Jubilaeumsfonds of the Austrian National Bank (OENB No. 15356) and the Austrian Science Fund (FWF, KLI544-B27) to LB-D.

## Conflict of interest

The authors declare that the research was conducted in the absence of any commercial or financial relationships that could be construed as a potential conflict of interest.

## Publisher’s note

All claims expressed in this article are solely those of the authors and do not necessarily represent those of their affiliated organizations, or those of the publisher, the editors and the reviewers. Any product that may be evaluated in this article, or claim that may be made by its manufacturer, is not guaranteed or endorsed by the publisher.
